# Supplementary material for: Wireless Passive Ceramic Sensor for Far-Field Temperature Measurement at High Temperatures
Source: Sensors (Basel). 2024 Feb 22;24(5):1407. doi: 10.3390/s24051407 (PMC10935068; doi:10.3390/s24051407)
Supplement: Supplementary file 1 [file sensors-24-01407-s001.zip › sensors-2872692-supplementary.pdf]

sensors

MDPI

Supplementary Information

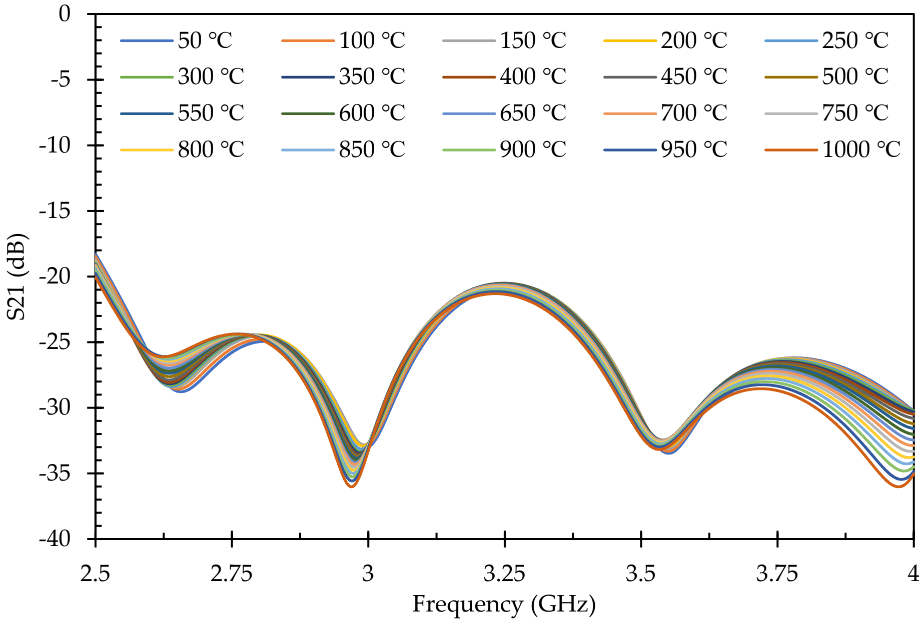

Figure S1. Frequency sweeps of the ITO normal ground plane sensor at 0.35 m

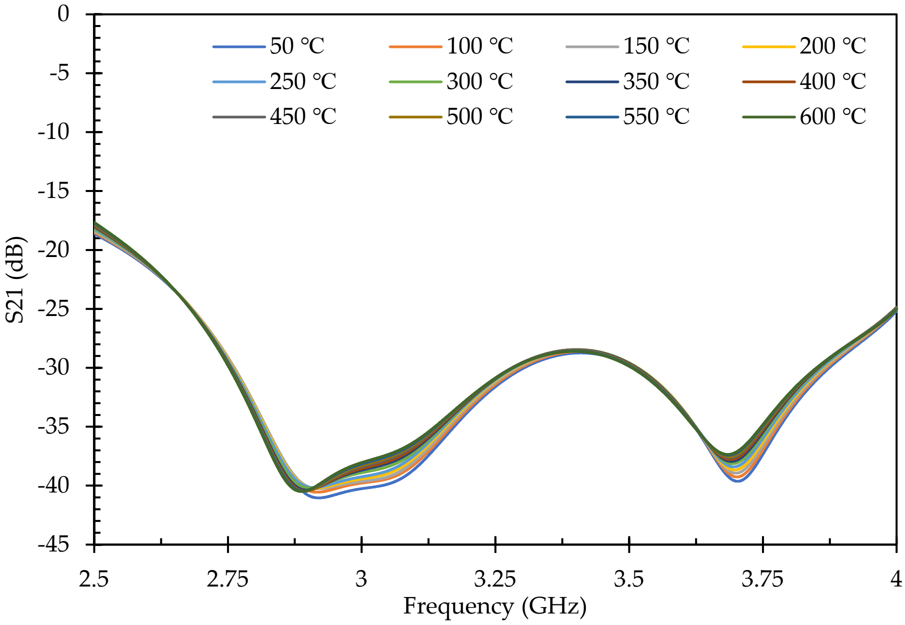

Figure S2. Frequency sweeps of the silver normal ground plane sensor at 0.50 m

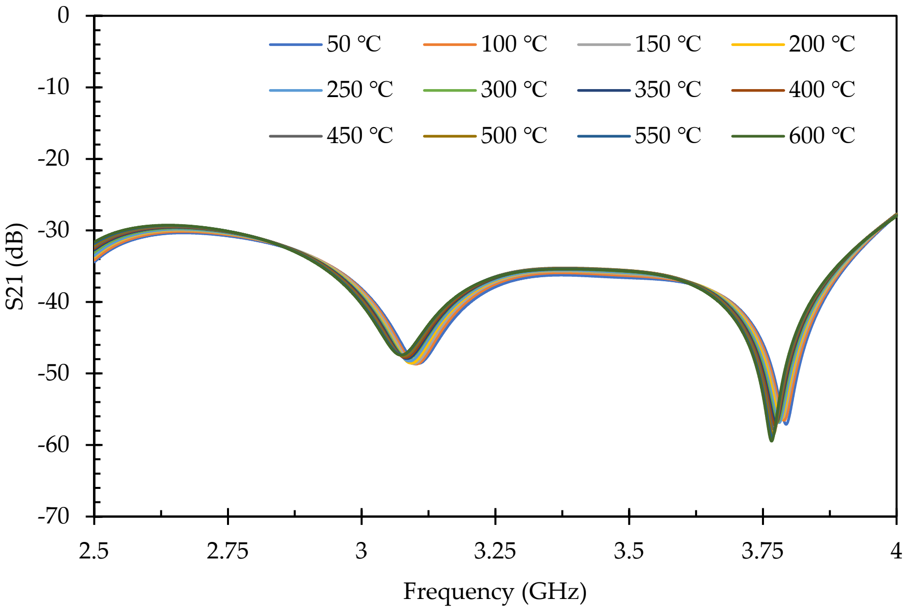

Figure S3. Frequency sweeps of the silver normal ground plane sensor at 0.75 m

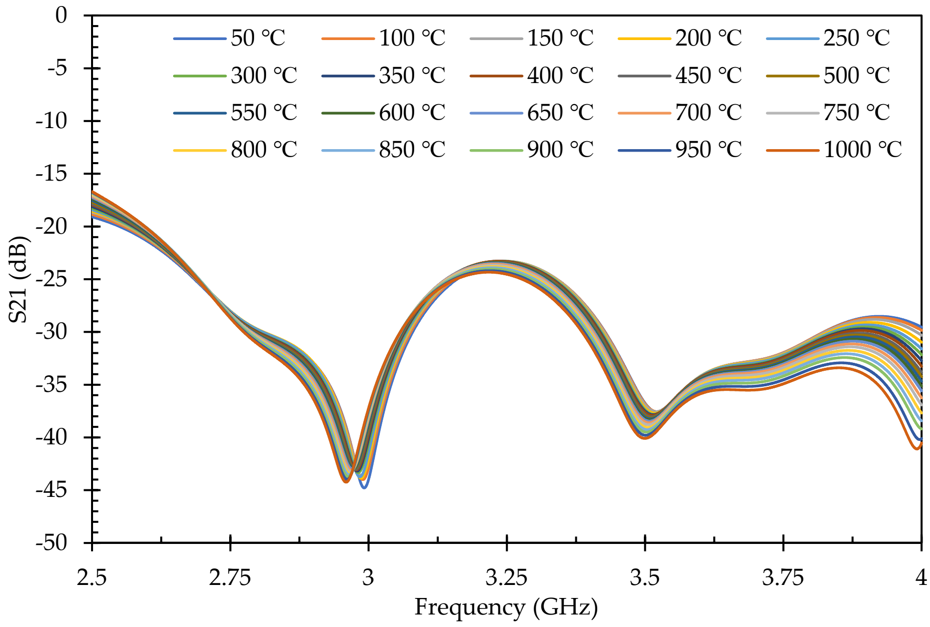

Figure S4. Frequency sweeps of the ITO normal ground plane sensor at 0.50 m

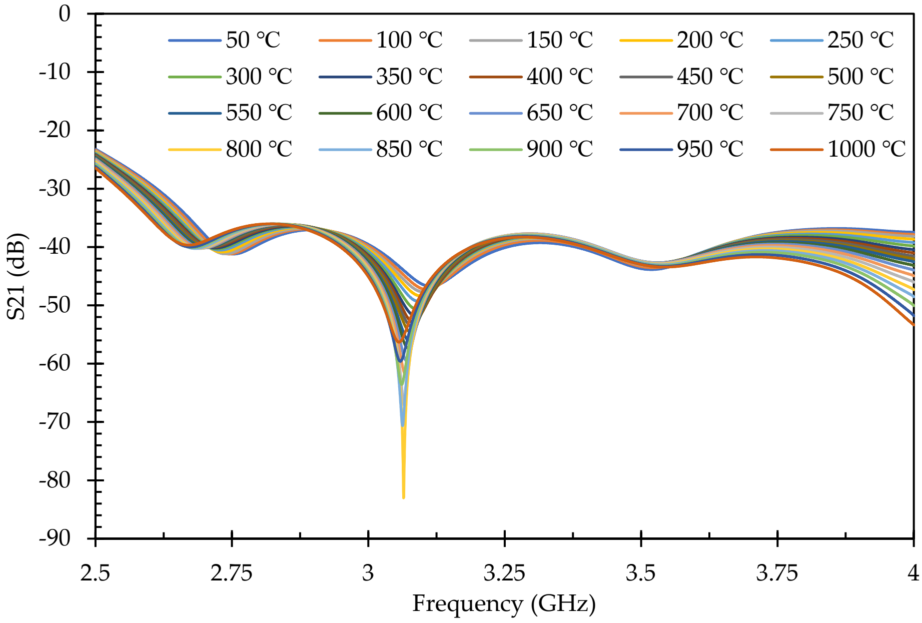

Figure S5. Frequency sweeps of the ITO normal ground plane sensor at 0.75 m

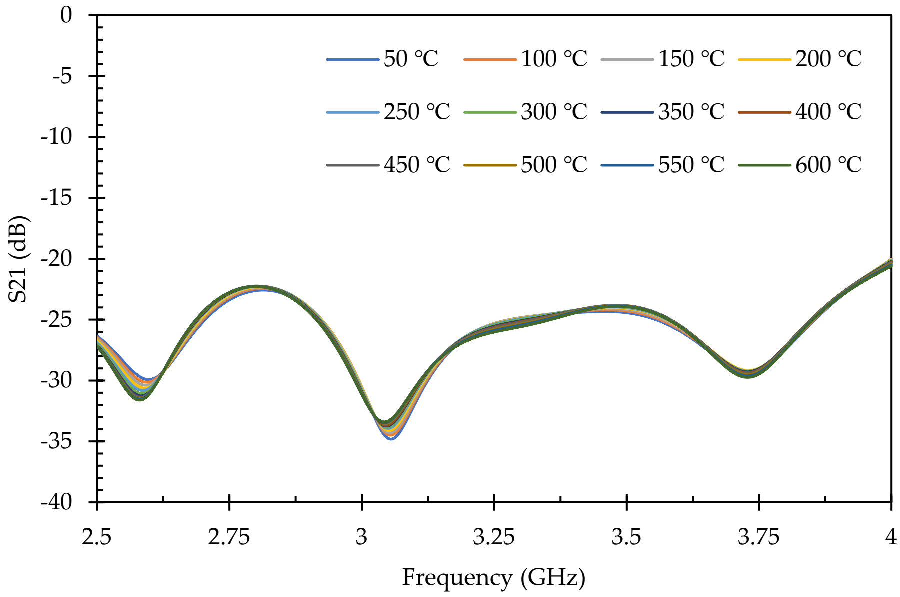

Figure S6. Frequency sweeps of the silver with RIS ground plane sensor at 0.35 m

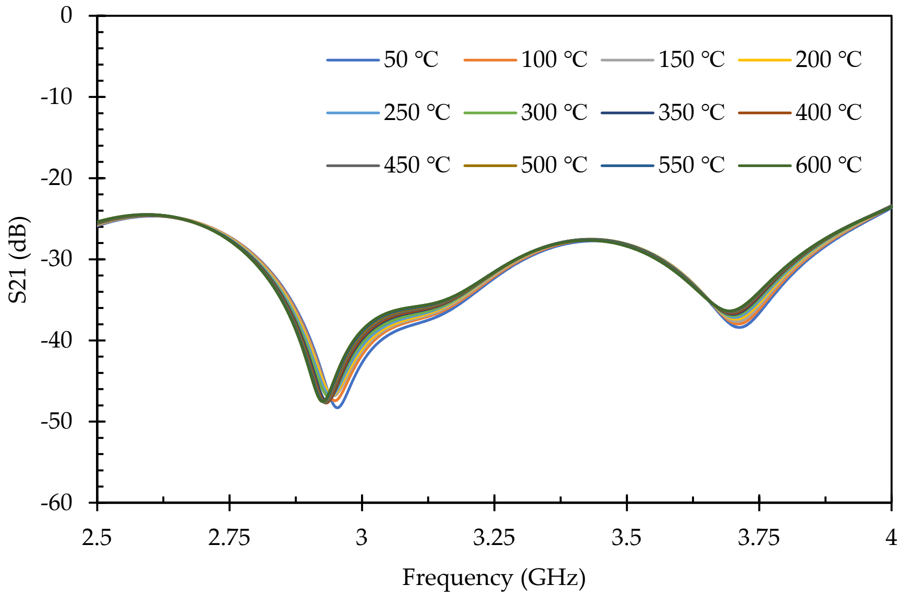

Figure S7. Frequency sweeps of the silver with RIS ground plane sensor at 0.50 m

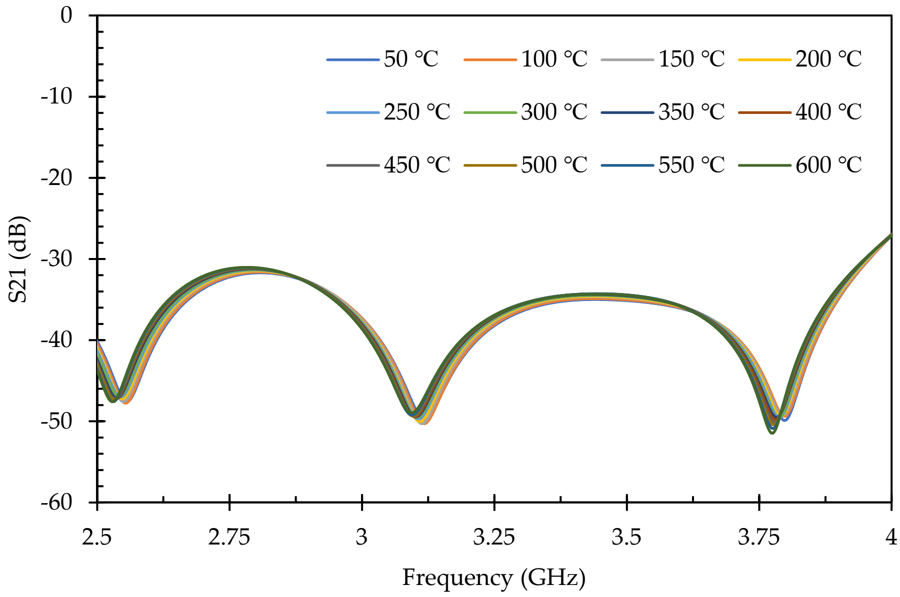

Figure S8. Frequency sweeps of the silver with RIS ground plane sensor at 0.75 m

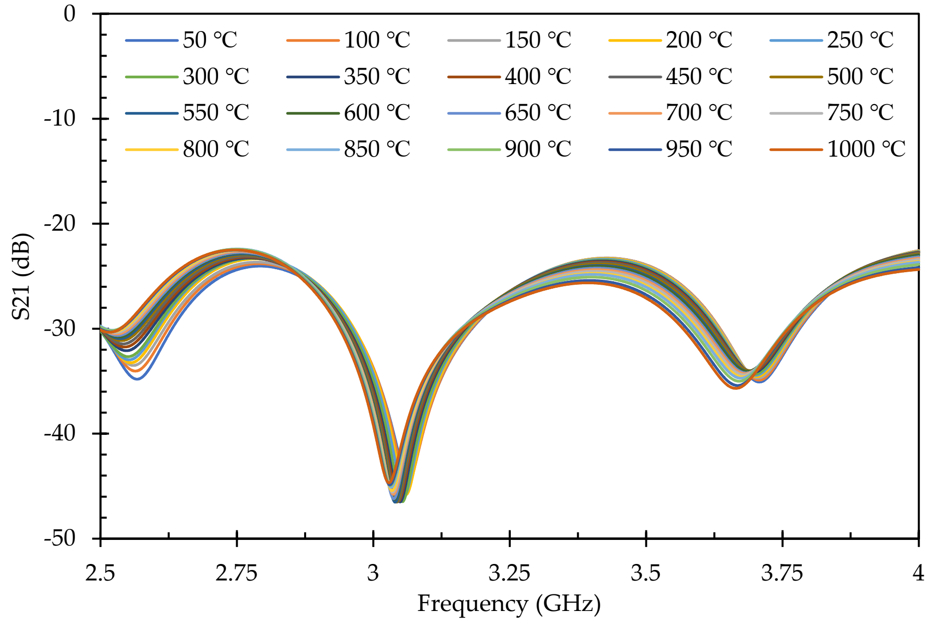

Figure S9. Frequency sweeps of the ITO with RIS ground plane sensor at 0.35 m

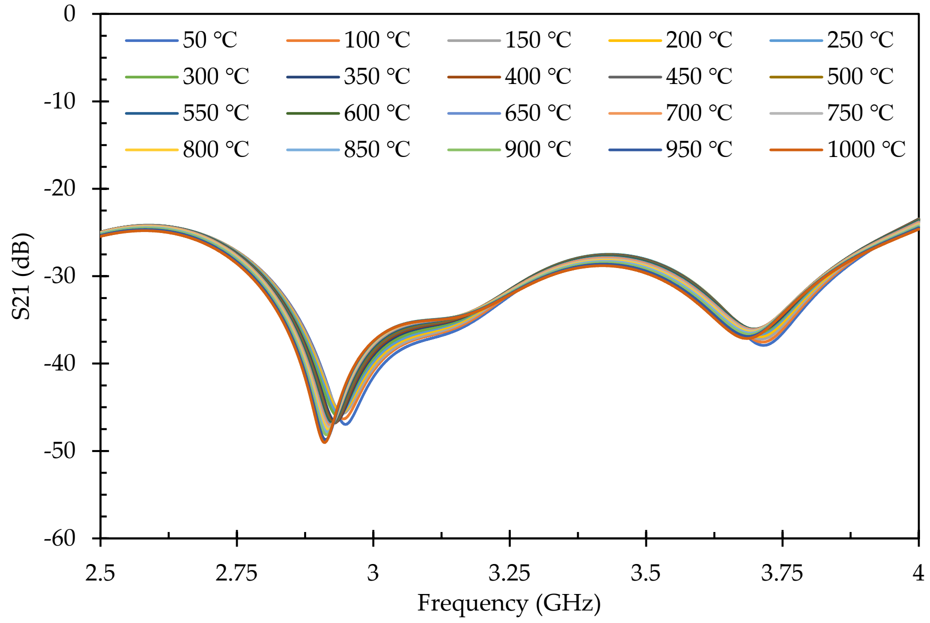

Figure S10. Frequency sweeps of the ITO with RIS ground plane sensor at 0.50 m

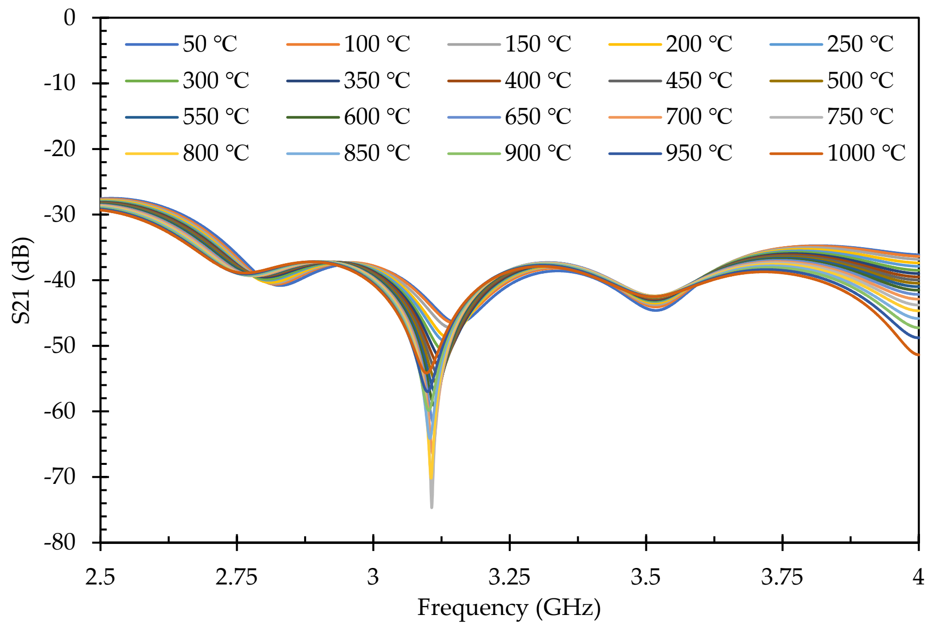

Figure S11. Frequency sweeps of the ITO with RIS ground plane sensor at 0.75 m
